# Accuracy of Doppler-Echocardiographic Mean Pulmonary Artery Pressure for Diagnosis of Pulmonary Hypertension

**DOI:** 10.1371/journal.pone.0015670

**Published:** 2010-12-17

**Authors:** Fikret Er, Stefan Ederer, Amir M. Nia, Evren Caglayan, Kristina M. Dahlem, Nasser Semmo, Natig Gassanov

**Affiliations:** 1 Department of Internal Medicine III, University of Cologne, Cologne, Germany; 2 Department of Medicine II, University Hospital Freiburg, Freiburg, Germany; Universidad Peruana Cayetano Heredia, Peru

## Abstract

**Background:**

The validity of Doppler echocardiographic (DE) measurement of systolic pulmonary artery pressure (sPAP) has been questioned. Recent studies suggest that mean pulmonary artery pressure (mPAP) might reflect more accurately the invasive pressures.

**Methodology/Principal Findings:**

241 patients were prospectively studied to evaluate the diagnostic accuracy of mPAP for the diagnosis of PH. Right heart catheterization (RHC) and DE were performed in 164 patients mainly for preoperative evaluation of heart valve dysfunction. The correlation between DE and RHC was better when mPAP (r = 0.93) and not sPAP (r = 0.81) was assessed. Bland-Altman analysis revealed a smaller variation of mPAP than sPAP. The following ROC analysis identified that a mPAP≥25.5 mmHg is useful for the diagnosis of PH. This value was validated in an independent cohort of patients (n = 50) with the suspicion of chronic-thromboembolic pulmonary hypertension. The calculated diagnostic accuracy was 98%, based on excellent sensitivity of 98% and specificity of 100%. The corresponding positive and negative predictive values were 100%, respectively 88%.

**Conclusion:**

mPAP has been found to be highly accurate for the initial diagnosis of PH. A cut-off value of 25.5 mmHg might be helpful to avoid unnecessary RHC and select patients in whom RHC might be beneficial.

## Introduction

Pulmonary hypertension (PH) is associated with restricted flow through the pulmonary circulation, increased pulmonary vascular resistance and right heart failure [1]. Since the last decades PH has been identified as a devastating disease with a high mortality in dependence on the clinical classifications [2], [3]. Early diagnosis is essential to identify patients at high risk, treat the PH and modify the etiologic substrate. PH has been defined as an elevation of mean pulmonary artery pressure (mPAP)≥25 mmHg in right heart catheterization (RHC) [1], [4]. When PH is suspected in patients based on the history, risk factor assessment, and physical examination, an echocardiogram has been addressed as the next appropriate study [4]. The Doppler echocardiogram (DE) can simultaneously provide an estimate of right ventricular systolic pressure (RVSP), functional and morphologic cardiac sequelae of PH, and identification of possible cardiac causes of PH or the presented clinical symptoms. The need for further invasive diagnostics is often triggered by the DE assessment of the peak systolic PAP (sPAP). But the reported accuracy of sPAP determination by DE is controversial. While initial comparisons between DE and RHC revealed an acceptable correlation [5], [6], recent studies questioned the diagnostic value of DE in PH [7]–[9]. In the present study we aimed to determine whether echocardiographic assessment of mPAP is more accurate than sPAP for initial diagnosis of PH and estimation of real pulmonary artery pressure.

## Methods

The study was performed in accordance with the STARD criteria to improve the quality of diagnostic accuracy [10]. The calculated diagnostic accuracy was validated in an independent cohort.

### Ethics Statement

The study complies with the Declaration of Helsinki. All participants gave their written informed consent and the Ethics Committee of the University of Cologne approved the conduct of this study.

### Subjects

Consecutive patients referred to the Cardiology Department of the University of Cologne were included in this prospective study from December 2008 to June 2010. All patients had a clinical indication for RHC due to heart failure, aortic or mitral valve dysfunction or the suspicion of PH. All patients in the validation group had a history of pulmonary embolism and the clinical suspicion of PH.

### Right Heart Catheterization

RHC was performed without sedation at rest in the cardiac catheter laboratory of the Cardiology Department of the University of Cologne. End-expiratory pressure measurements were taken from the right atrium, right ventricle, pulmonary artery and pulmonary capillary.

### Transthoracic echocardiography

Comprehensive two-dimensional echocardiography was performed in all patients within 120 minutes before right heart catheterization using a Phillips iE33 ultrasound device equipped with a standard transducer operating at 1–5 MHz without using saline contrast. The echocardiography was performed by three different cardiologists. In each patient only one cardiologist performed the examination, randomly. Multiple views were recorded to identify optimal view for analysis as recommended in actual guidelines [11]–[13]. The right atrial pressure (RAP) was estimated by evaluating the inferior vena cava (IVC) diameter (IVCd) and change with respiration [14], [15]: when IVCd was less than 20 mm and the collapsibility greater than 50% RAP was estimated to be 5 mmHg versus 10 mmHg when the collapsibility was less than 50%. When the IVCd was greater than 20 mm RAP was estimated to be 15 mmHg when the collapsibility was greater than 50% and to be 20 mmHg when the collapsibility was less than 50%.

Additionally to the mean gradient estimation, mPAP was calculated using the Chemla formula (mPAP = 0.61×sPAP +2 mmHg) and the Syyed formula (mPAP = 0.65×sPAP +0.55 mmHg) [16]–[18].

Continuous wave Doppler was used to determine the peak velocity of the tricuspid regurgitant (TR) jet at end-expiration. Patients were excluded when TR jet was not available. The highest TR velocity was measured and traced to obtain the peak and mean systolic right-ventricular-right-atrial (RV-RA) gradient. The mean gradient was calculated by tracing the TR time-velocity integral plus RAP [19]. The sPAP was calculated using the highest RV-RA gradient plus estimated RA pressure. The mPAP was calculated as mean RV-RA pressure plus estimated RA pressure. The quality of continuous Doppler envelope was graded by a blinded cardiologist from 1 (excellent visualization with full spade shaped Doppler envelope with exactly detectable peak) to 5 (poorly visualization of Doppler signal and peak velocity).

The potential confounding factors in echocardiographic right heart assessment like right heart dimensions, the presence of atrial fibrillation or severe tricuspid regurgitation were documented, but not corrected in any direction. In these cases the measurements were performed like in all other patients.

The right ventricular function was estimated by calculating the tricuspid annular plane systolic excursion (TAPSE). Left ventricular ejection fraction (LVEF) was estimated by the Simpson's rule in the four and two chamber views.

### Statistics

All variables were tested for normal distribution with the Kolmogorov-Smirnov test. Continuous variables are expressed as means ± standard deviation (SD). Comparison of 2 means was performed with the t test for normally distributed variables and the Mann-Whitney U test for non-Gaussian variables. Chi-square test was used for nonparametric comparisons. For diagnostic utility calculations receiver operating characteristic (ROC) curves were used. Results are expressed in terms of area under the curve (AUC) and 95% CI for this area. Sensitivity and specificity were estimated with ROC curves. Accuracy, positive predictive value (PPV) and negative predictive value (NPV) were calculated accordingly. Pressure comparisons were done using analysis described by Bland-Altman with predefined accuracy as 95% limits of agreement ±2xSD [20]. All statistical tests were 2-tailed, and p<0.05 was considered statistically significant. Statistical analysis was performed using SPSS 18 (SPSS GmbH Software – IBM Company, Munich, Germany).

## Results

### Baseline characteristics

191 consecutive patients with an indication for RHC were eligible for the study. 7 patients refused to participate in the study, in 9 patients echocardiography could not be performed within the predefined time schedule and 11 in patients an analyzable TR jet was not available. Data from a total of 164 patients were available for final analysis. The baseline characteristics of the study participants are displayed in Table 1. The majority of the patients underwent RHC for invasive evaluation of the aortic (n = 74) or mitral (n = 49) valve dysfunction. In 41 patients RHC was performed for heart failure assessment, in 11 of these patients for scheduling for heart transplantation.

**Table 1:** Characteristics of 164 patients and indications for RHC.

**Age**	63.7±15.5
**Men (%)**	88 (54)
**BMI**	26.6±5.5
**Echocardiography**	
Left atrial diameter, mm	43.6±10.3
Left ventricular enddiastolic diameter, mm	51.7±11.2
Left ventricular ejection fraction, %	54.9±14.5
Enddiastolic interventricular septum, mm	11.2±2.3
TAPSE, mm	18.1±4.6
Right mid-ventricular diameter, mm	36.4±7.5
Right ventricular diameter-long axis, mm	60.74±14.8
mPAP, mean gradient method (mmHg)	37.1±12.2
mPAP, calculated with Chemla formula (mmHg)	37.9±12.5
mPAP, calculated with Syyed formula (mmHg)	37.5±11.8
**Medical history**	
Hypertension (%)	87 (53)
Coronary heart disease (%)	65 (40)
Diabetes (%)	27 (16)
Heart failure (%)	95 (58)
Ischemic cardiomyopathy (%)	32 (20)
Dilated cardiomyopathy (%)	41 (25)
Chronic obstructive pulmonary disease (%)	11 (7)
Atrial fibrillation (%)	42 (26)
**NYHA functional class**	
I (%)	8 (5)
II (%)	61 (37)
III (%)	83 (51)
IV (%)	12 (7)
**Indication for RHC**	
Aortic valve evaluation (%)	41 (25)
- Aortic valve stenosis (%)	37 (23)
- Aortic valve regurgitation (%)	4 (2)
Mitral valve evaluation (%)	74 (45)
- Mitral valve stenosis (%)	11 (7)
- Mitral valve regurgitation (%)	63 (38)
Heart failure evaluation (%)	49 (30)
- Evaluation for heart transplantation (%)	11 (7)

BMI indicates body mass index (kg/m^2^), RHC indicates right heart catheterization.

### Correlation of invasive versus echocardiographic sPAP, mPAP and RAP

**Figure 1:**
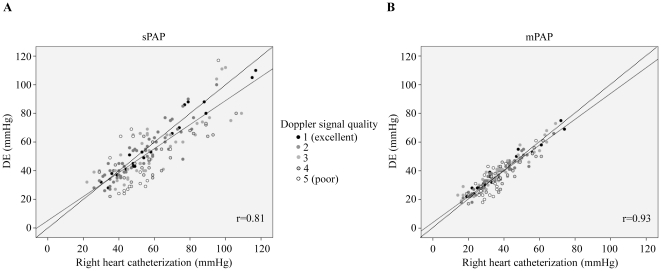
DE versus RHC correlations. DE mPAP (B) was better correlated with RHC than sPAP (A). Dotted lines mark virtual best correlation of 1 and solid lines mark the real correlation. r indicates the correlation coefficient, sPAP indicates systolic pulmonary artery pressure.

In all patients echocardiographic evaluation of mPAP and sPAP were performed. The comparison of invasive versus echocardiographic mPAP revealed a better correlation than the sPAP in RHC versus echocardiography (Figure 1). The correlation coefficient for DE and invasive sPAP was 0.81 (p<0.001) compared to 0.93 for mPAP in DE versus RHC (p<0.001). Both, echocardiographic sPAP and mPAP calculations were influenced by the estimated RAP. The correlation of DE and invasive RAP was weaker than seen for mPAP and sPAP (r = 0.67; p<0.001). Better Doppler signal quality improved the documented correlations (Figure 1).

**Figure 2:**
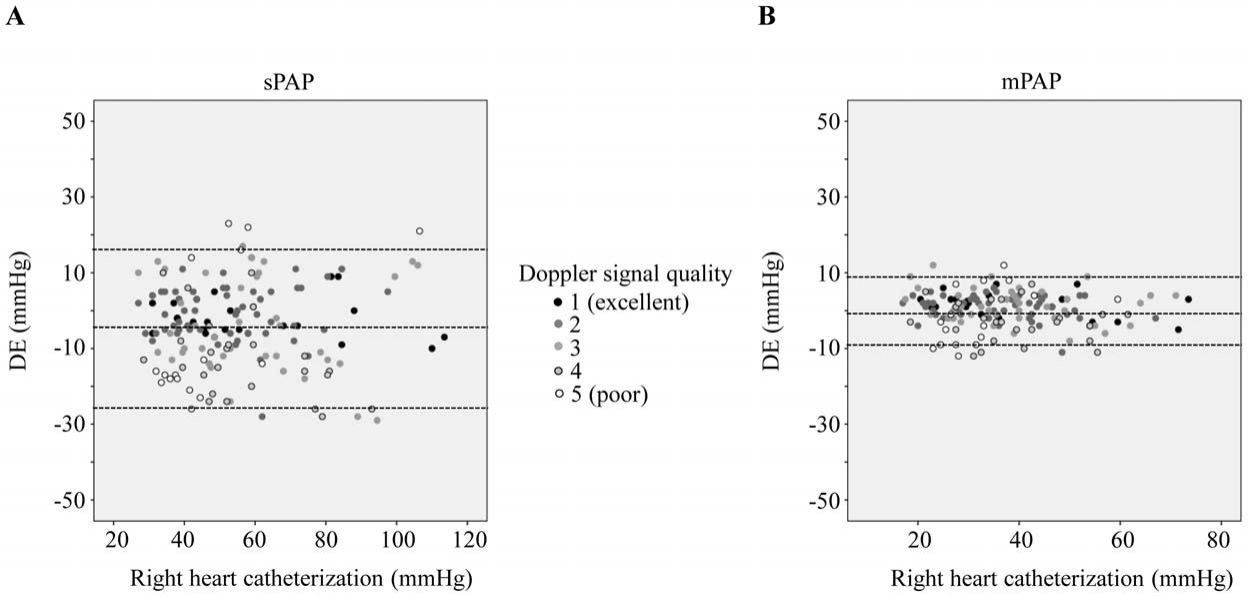
Bland-Altman plot of DE estimates of PA and RHC pressures for sPAP (A) and mPAP (B). Smaller bias and limits of agreement present in mPAP measurements compared to sPAP measurements.

**Figure 3:**
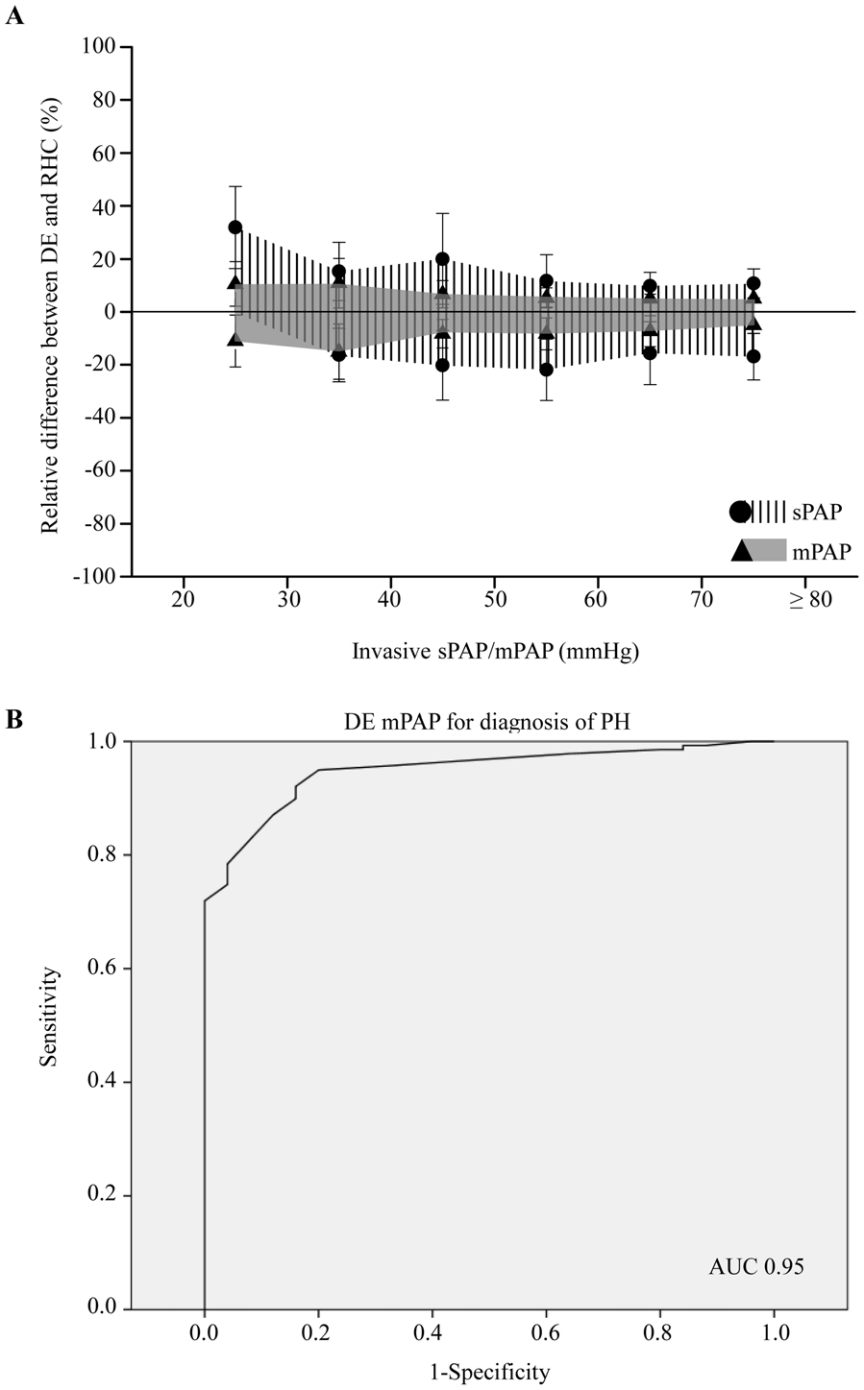
Analysis of relative differences and ROC analysis of mPAP for diagnosis of PH. A, the relative positive and negative deviation between DE and RHC were larger for sPAP than mPAP. B, ROC analysis reveal an excellent diagnostic accuracy of mPAP for the diagnosis of PH with an area under the curve (AUC) of 0.95.

Using Bland-Altman analysis, the bias for the echocardiographic estimates of sPAP was -4.1 mmHg with 95% limits of agreement ranging from +23 mmHg to −29 mmHg (Figure 2A). In contrast bias for the mPAP measurements was 0.3 mmHg with 95% limits of agreement ranging from +12 to −12 mmHg (Figure 2B). Absolute values of mPAP lower than sPAP values, which could explain smaller variations of mPAP. To rule out this fact the relative variation of mPAP and sPAP in DE vs. RHC were calculated. But even the relative differences between DE and RHC were larger for sPAP than mPAP indicating a better correlation of DE mPAP and RHC vs. DE sPAP and RHC (Figure 3A).

Correlation and Bland-Altman analysis identified an excellent correlation of echocardiographic mPAP and RHC between 20 and 40 mmHg (Figure 1). We identified this range to be important for the diagnosis of PH. Therefore using the ROC analysis we tested the usefulness of echocardiographic mPAP for the diagnosis of PH. For this calculation PH was defined as invasive mPAP≥25 mmHg [21]. The ROC analysis revealed a useful sensitivity and specificity of mPAP for diagnosis of PH with DE, reflected by an area under curve (AUC) of 0.95 (95 CI; 0.914–0.983; p<0.001; Figure 3B). A cut-off value of mPAP≥25.5 mmHg could detect PH with a sensitivity of 92% and a specificity of 84%. The corresponding likelihood ratios (LR) were 5.76 for positive LR and 0.18 for negative LR (Table 2).

**Table 2:** Diagnostic value of different mPAP cut-offs for diagnosis of PH.

mPAP≥ (mmHg)	Sensitivity	Specificity	Positive LR	Negative LR
23.5	96	68	2.99	0.33
24.5	95	80	4.75	0.21
**25.5**	**92**	**84**	**5.76**	**0.18**
26.5	90	84	5.62	0.18
27.5	87	88	7.26	0.14

LR indicates likelihood ratio.

### Validation of mPAP for the Diagnosis of PH

**Figure 4:**
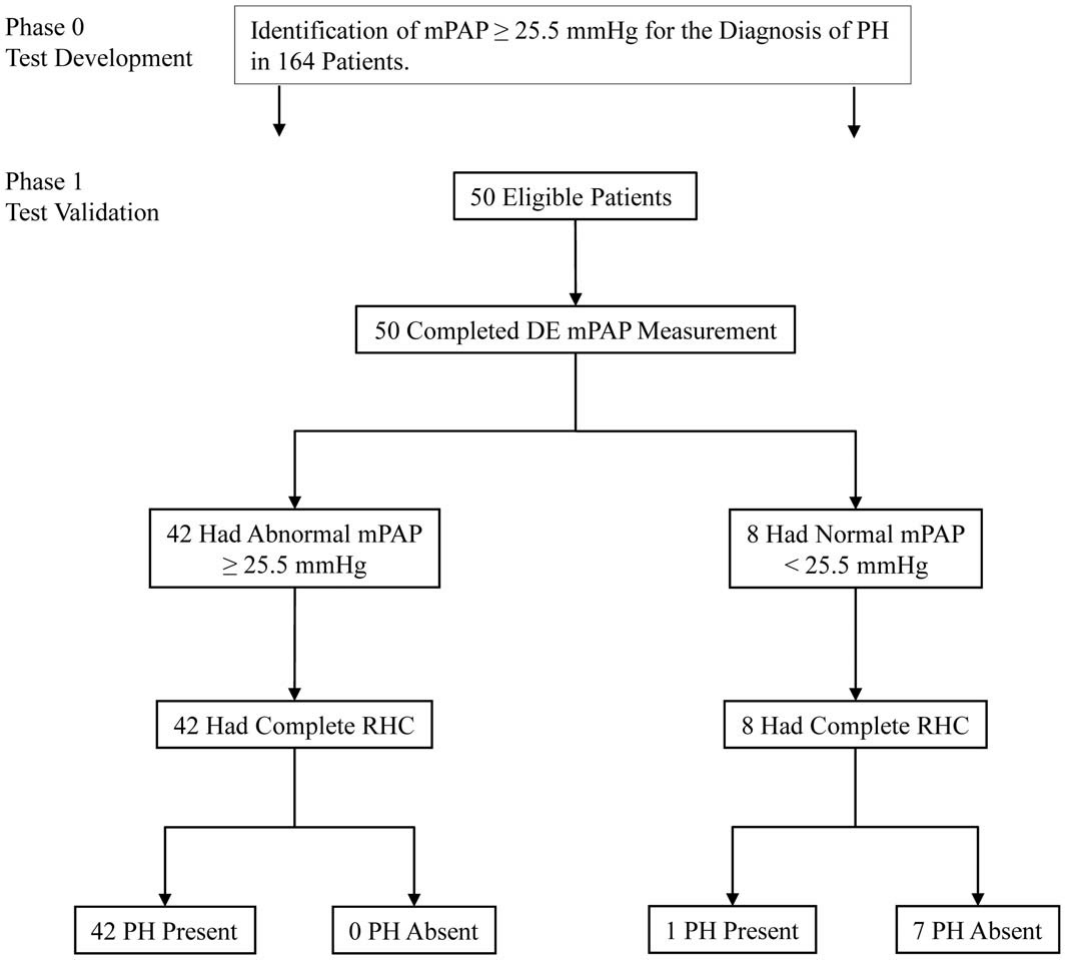
Study flowchart in accordance to the STARD criteria. In phase 0 diagnostic criteria were evaluated in 164 patients undergoing DE and RHC. In phase 1 the calculated cut-off value for mPAP was validated in a cohort of patients with the suspicion of PH.

The diagnostic accuracy of mPAP for the detection of patients with PH was tested in an independent cohort of patients. 50 consecutive patients who were referred to the Cardiology Department for invasive RHC with a history of pulmonary embolism and the clinical suspicion of chronic-thromboembolic PH were included. The demographics and invasive vs. DE pressures are displayed in table 3. There were no exclusions. Using the DE cut-off value of 25.5 mmHg 42 patients were identified to have a PH (Figure 4). RHC confirmed in all 42 patients the diagnosis of PH. In 8 patients DE suggested that a PH could be excluded. In one of these patients the result was false negative. The calculated diagnostic accuracy was 98%, based on excellent sensitivity of 98% and specificity of 100%. The corresponding positive and negative predictive values were 100% and 88%.

**Table 3:** Demographics, RHC and DE measurements of the validation group (n = 50).

**Age**	66.9±14.5
**Men (%)**	17 (34)
**BMI**	27.1±5.1
**WHO functional class**	
I (%)	7 (14)
II (%)	13 (26)
III (%)	26 (52)
IV (%)	4 (8)
**Invasive testing**	
sPAP, mmHg (DE)	63.6±23.6 (62.3±25.7)
mPAP, mmHg (DE)	39.5±14.9 (40.3±14.4)
mPAP 25–36 mmHg, mild PH (%)	17/43 (40)
mPAP 37–49 mmHg, moderate PH (%)	14/43 (33)
mPAP≥50 mmHg, severe PH (%)	12/43 (28)
PCWP, mmHg	9.9±4.4
PVR, dynesXsecXcm^−5^	598±143
SVR, dynesXsecXcm^−5^	1230±317
Cardiac output (L/min)	3.6±1.2
PA SO_2_ (%)	65±11.3
Aorta SO_2_ (%)	94±4.9
**Final Dana Point classification of PH**	
1. Pulmonary arterial hypertension (%)	2/43 (5)
2. PH due to left heart failure (%)	7/43 (16)
3. PH due to lung diseases (%)	1/43 (2)
4. Chronic thromboembolic hypertension (%)	29/43 (67)
5. PH with unclear and/or multifactorial mechanisms (%)	4/43 (9)

BMI indicates body mass index (kg/m^2^), RHC indicates right heart catheterization, PCWP indicates pulmonary capillary wedge pressure, PVR indicates pulmonary vascular resistance. SVR indicates systemic vascular resistance, PA SO_2_ indicates pulmonary artery oxygen saturation, aorta SO_2_ indicates aortic oxygen saturation.

## Discussion

We examined the diagnostic accuracy of mPAP for the diagnosis of PH. We applied DE assessment in a large number of patients undergoing RHC for several reasons. Comparison analysis revealed that DE mPAP reflects more precisely the invasive pressures than DE sPAP does. Based on this observation the performed ROC analysis displayed that mPAP is useful for diagnosis of PH. The accuracy of mPAP for PH was validated in an independent high-risk cohort of patients with the suspicion of PH. An excellent sensitivity of 98%, specificity of 100% and accuracy of 98% of this diagnostic tool could be confirmed. Only in one of forty-three patients with borderline PH DE was false negative and in none of the patients false positive.

Despite of promising therapeutic options emerged in the last decades, mortality remains high among patients with PH [22], [23]. Early diagnosis of PH may change its detrimental character and its annual mortality rate of 15% [24]. But still the diagnosis of PH is challenging. The invasive assessment of the right heart and pulmonary arteries has been established as the gold standard in the diagnosis of PH [21]. It helps to differentiate pre- and postcapillary PH. Due to its invasive character RHC is not useful as a routine screening method. Echocardiography is a widely available and accepted noninvasive diagnostic instrument for assessment of right heart function and dimension [13], [25]–[29]. Initial studies reported a good correlation between DE and RHC [5], [6].

But recent DE studies questioned the diagnostic value of DE for PH assessment displaying a large variation of sPAP [7]–[9]. In a well-designed study Fisher and Colleagues assessed the usability of DE for evaluation of sPAP and found despite a good correlation a wide and inacceptable discrepancies between DE and RHC in Bland-Altman analysis [7]. Our results are in agreement with previous findings. Therefore DE sPAP may not useful for the diagnosis and estimation of pulmonary artery pressure.

It is of clinical interest to identify patients with PH early and in a feasible and economic way. We identified DE as a helpful tool for the diagnosis of PH when mPAP and not sPAP is used. Our results concerning the correlation of mPAP in DE vs. RHC are supported by recent studies [30], [31]. Aduen and coworkers identified echocardiographic mPAP as a valuable parameter for the assessment of pulmonary artery pressure and confirmed that the method used in our study of mean gradient estimation is equally applicable to the Chemla formula and the Syyed formula [16]–[18], [30], [31].

In 43 of 50 patients with the suspicion of PH the diagnosis of PH could be confirmed. Of these patients almost one third had mild, one third moderate and one third severe PH. Hence, echocardiographic mPAP may be suitable in a wide range of PH severity. Especially the detection of PH in mildly symptomatic or mildly elevated mPAP is crucial. Echocardiographic mPAP may be a helpful screening tool in these patients.

In conclusion we identified the echocardiographic mPAP measurement as accurate for diagnosis of PH. mPAP measurement may prevent unnecessary RHC and identify patients at high risk for PH.

### Study limitations

This study is limited by its focus on a single center's experience and the limited sample size of the validation group. In almost 6% of the patients a TR jet was not analyzable. Saline contrast may increase the rate on available TR jet signals. The estimation of RAP is generally challenging as in our study. But despite the large variation in RAP the calculated accuracy of mPAP was excellent, suggesting that TR signal alone reflects accurately the PAP. RHC and DE were not performed simultaneously. While during Swan-Ganz catheterization simultaneous measurements may be suitable, it's a technical challenge performing echocardiography in the catheter lab during RHC.
